# Tocotrienol-Rich Fraction Attenuates Blue Light-Induced Oxidative Stress and Melanogenesis in B16-F1 Melanocytes via Anti-Oxidative and Anti-Tyrosinase Properties

**DOI:** 10.3390/ijms242015373

**Published:** 2023-10-19

**Authors:** Juvenia Rui En Neo, Cheryl Wei Ling Teo, Yee Wei Ung, Wei Ney Yap

**Affiliations:** 1 Research and Development Department, Davos Life Science, 3 Biopolis Drive, #04-19 Synapse, Singapore 138623, Singapore; ruien.neo@davoslife.com (J.R.E.N.);; 2 Research and Development Department, KL-Kepong Oleomas (KLK Oleo), Level 8, Menara KLK, No 1, Jalan PJU 7/6, Mutiara Damansara, Petaling Jaya 47810, Malaysia; yw.ung@klkoleo.com

**Keywords:** blue light, oxidative stress, pigmentation, tocotrienol-rich fraction, anti-oxidant, anti-melanogenesis, melanocyte

## Abstract

Our skin is constantly exposed to blue light (BL), which is abundant in sunlight and emitted by digital devices. Prolonged exposure to BL can lead to oxidative stress-induced damages and skin hyperpigmentation. For this study, we used a cell line-based model to examine the protective effects of tocotrienol-rich fraction (TRF) on BL-induced oxidative stress and hyperpigmentation in B16-F1 melanocytes. Alpha-tocopherol (αTP) was used as a comparator. Molecular assays such as cell viability assay, flow cytometry, western blotting, fluorescence imaging, melanin and tyrosinase analysis were performed. Our results showed that TRF effectively suppressed the formation of reactive oxygen species and preserved the mitochondrial membrane potential. Additionally, TRF exhibited anti-apoptotic properties by reducing the activation of the p38 mitogen-activated protein kinase molecule and downregulating the expression of cleaved caspase-3. Moreover, TRF modulated tyrosinase activity, resulting in a lowered rate of melanogenesis and reduced melanin production. In contrast, αTP did not exhibit significant protective effects against skin damages and pigmentation in BL-induced B16-F1 cells. Therefore, this study indicates that TRF may offer superior protective effects over αTP against the effects of BL on melanocytes. These findings demonstrate the potential of TRF as a protective natural ingredient that acts against BL-induced skin damages and hyperpigmentation via its anti-oxidative and anti-melanogenic properties.

## 1. Introduction

Our skin acts as a crucial barrier between the body and the external environment, protecting it from various environmental factors such as solar radiation and air pollution [1]. It serves as the primary defence and repair mechanism for the body while also contributing to the maintenance of normal homeostasis. It is made up of three layers: the epidermis, dermis, and subcutaneous tissue. Melanocytes, which are located in the basal layer of the epidermis, are an integral type of skin cell. These specialized cells synthesize melanin through a complex enzymatic process that gives rise to skin, eye, and hair colour in humans and animals [2,3]. Sunlight, which is essential for the survival of most living organisms, consists of a range of wavelengths. The solar spectrum includes ultra-violet (UV) rays, visible light, and infrared rays, that enter the terrestrial environment. Among these, UV rays comprise only 2% of the solar spectrum, while visible light and infrared rays account for nearly half of the solar spectrum [4]. Numerous studies have focused on understanding the skin protective measures required to fight against UV rays, as they have the shortest wavelength (100 to 400 nm) and more energy compared to visible light (400 to 780 nm) and infrared rays (780 to 3000 nm), which have longer wavelengths and less energy [5]. Despite having less energy, visible light is known to penetrate deeper into the skin due to its longer wavelength. The visible light spectrum consists of various colours, including (increasing order of wavelengths) violet, blue, green, yellow, orange, and red light. Blue light (BL; 450 to 485 nm) is one of the most extensively studied visible light wavelengths [4]. BL can be found in sunlight and artificially in devices such as light-emitting diode (LED) screens (laptop, mobile, and tablet screens) or compact fluorescent lamp devices [6]. Recent studies have shown that BL can have detrimental effects on the skin, including the induction of skin pigmentation and photoaging [7,8]. BL has also been extensively studied for its potential in phototherapy against skin diseases such as melanoma as it can slow cell growth and promote apoptosis in melanoma cells. These effects are attributed to the accumulation of reactive oxygen species (ROS) molecules such as superoxide, which can lead to mitochondrial membrane alterations and cell death [9,10]. As such, prolonged exposure to BL on healthy skin may result in irreversible damage to the skin. Therefore, it is widely acknowledged that exposure to artificial BL in modern times may have detrimental effects on skin health, leading to oxidative stress and pigmentation. Consequently, further studies are necessary to investigate the measures for protecting melanocytes against the damaging effects of BL.

In such circumstances, anti-oxidants can effectively prevent damage caused by oxidative stress by maintaining a balanced cellular redox environment [11]. Vitamin E has a well-established history of use in dermatology, cardiovascular health, and neuroprotection due to its potent anti-oxidant and anti-inflammatory properties. It can be found in various food sources, such as plant oils and seeds [12]. Studies have shown that vitamin E can alleviate inflammatory skin diseases and provide significant photoprotective effects [13,14]. Vitamin E primarily exists in eight isoforms, including α-, β-, γ-, and δ-tocopherol (TP) and α-, β-, γ-, and δ-tocotrienol (T3). Both TPs and T3s differ in terms of their structural form, where T3s have the presence of an unsaturated side chain that enables better penetration into fatty tissues, making them more potent anti-oxidants compared to TPs [15]. T3s have also been shown to protect the skin from oxidative stress-induced damage caused by particulate matter and exhibit whitening effect by suppressing tyrosinase activity in UV-induced melanocytes, as reported in our previous studies [16,17]. In this study, we investigated the protective effects of T3s against the harmful effects of prolonged BL exposure on murine melanoma cells B16-F1. Our study involved using tocotrienol-rich fraction (TRF), which comprises four T3 isoforms (α-, β-, γ-, and δ-T3s) and αTP. The other vitamin E comparator used in this study was αTP, a commonly abundant form of vitamin E in the body [15]. Although several studies have investigated the benefits of T3s for skin health, there is a lack of studies specifically focused on the effects of T3s on prolonged BL exposure. Given the potent anti-oxidative activities of T3s, it is hypothesized that T3s can protect the skin from excessive ROS generation, thereby alleviating cell death and skin hyperpigmentation. Therefore, our study aimed to elucidate the protective effects of T3s against BL exposure by investigating the anti-oxidative and anti-pigmentating mechanisms. To achieve this, the effects of TRF on BL-induced melanocytes were examined through a cell viability assay. Subsequent experiments revealed the presence of apoptosis, ROS formation, mitochondria damage, an increase in melanin levels, and elevated tyrosinase activity induced by BL. Additionally, an upregulation of activated p38 mitogen-activated protein kinases (MAPK) and cleaved caspase-3 protein were observed. Collectively, these results illustrate the protective abilities of TRF against BL-induced damage and pigmentation in melanocytes.

## 2. Results

### 2.1. TRF Improved Cell Viability of BL-Induced Melanocytes

**Figure 1:**
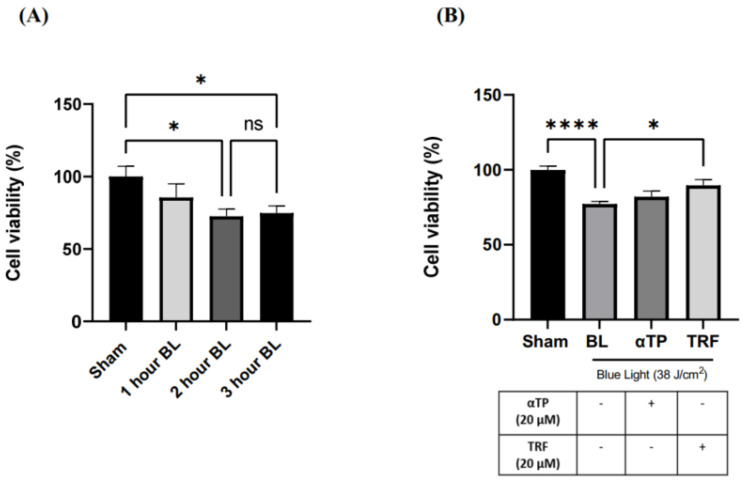
Cell viability of B16-F1 was evaluated via MTT assay 24 h after BL exposure and treatment. (**A**) Measurement of B16-F1 cell viability exposed with different dosages of BL. (**B**) Measurement of B16-F1 cell viability induced with BL, accompanied with αTP or TRF treatment. Data shown are expressed as % of sham and expressed as mean ± standard error of the mean (SEM). **** *p* < 0.0001 and * *p* < 0.05 indicate statistical significance. ns indicates no statistical significance.

The 3-[4,5-dimethylthiazol-2-yl]-2,5-diphenyltetrazoliumbromide (MTT) assay was used to assess cell proliferation. To determine the optimal dosage of BL exposure for the cells, we performed cell irradiation at three different time points—1 h (12 J/cm^2^), 2 h (25 J/cm^2^), and 3 h (38 J/cm^2^)—using an MTT assay. Our results show a dose-dependent decrease in cell viability, with no significant difference between 25 and 38 J/cm^2^ (Figure 1A). Thus, 38 J/cm^2^ was selected for our experiment to better represent real-life BL exposure. This dosage is equivalent to approximately 300 h of BL exposure from electronic devices at 100% screen brightness [18]. Additionally, a previous study indicated that this dosage induced minimal lethal oxidative stress in cells, making it suitable for accurate experimentation [7]. We further assessed the effect of TRF on cell viability and found that treatment with 20 µM of TRF significantly increased the cell viability of BL-treated B16-F1 cells compared to αTP, where only a modest increase was observed (Figure 1B).

### 2.2. TRF Inhibited BL-Induced Cell Apoptosis

**Figure 2:**
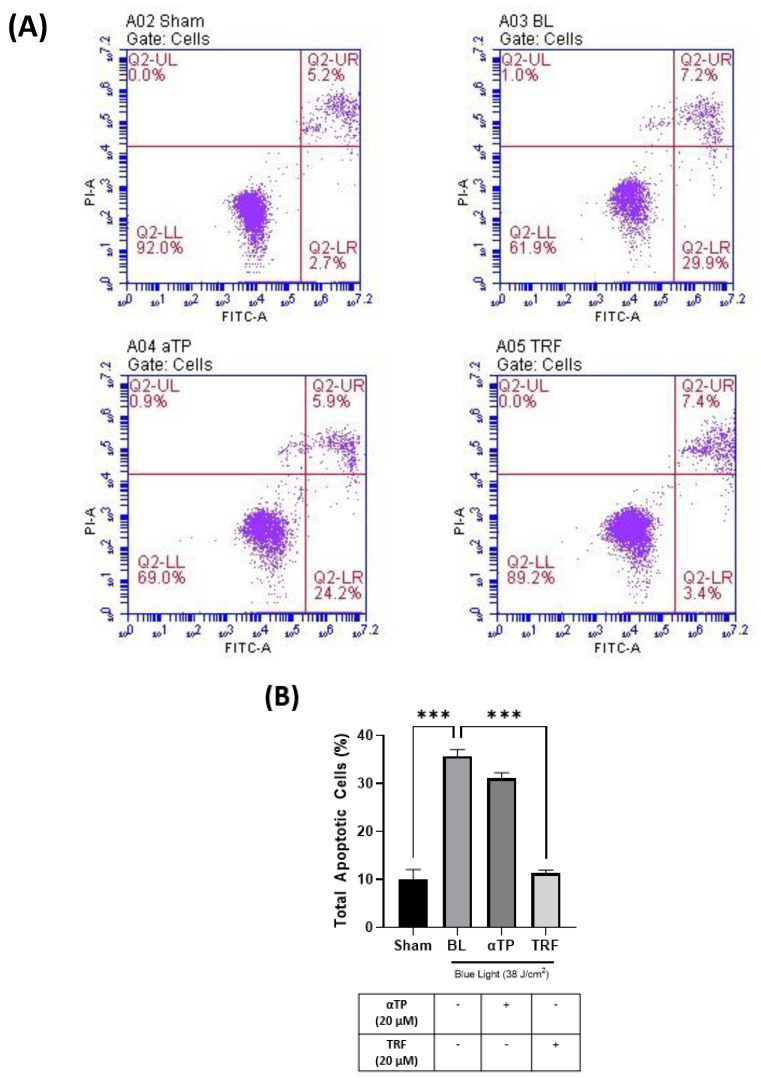
Flow cytometry assay of B16-F1 cells induced with BL and treatment. (**A**) Population of healthy (Q2-LL), early apoptotic (Q2-LR), late apoptotic (Q2-UR), and necrotic cells (Q2-UL) in BL-induced B16-F1 cells with designated treatment. (**B**) Total apoptotic B16-F1 cells as measured based on the early and late apoptotic populations. Data shown are expressed as % of sham and expressed as mean ± SEM. *** *p* < 0.001 indicates statistical significance.

Based on the findings presented in Section 2.1, our study aimed to investigate the level of apoptosis in B16-F1 cells following exposure to blue light. To provide further evidence supporting our findings, we conducted a flow cytometry assay to assess the population of apoptotic cells in both BL-induced/treated groups. A previous study showed that BL induced cellular apoptosis as observed in the B16-F10 cells [9]. In our study, we used an Annexin V/Propidium Iodide (PI) double-staining assay to measure the population of apoptotic and necrotic cells. Our results showed a significant increase in the population of total apoptotic cells, particularly in the early apoptotic group, in the BL-induced melanocytes (Figure 2A). Notably, the cells treated with TRF exhibited a rescue effect, as the number of apoptotic cells were significantly lower compared to the BL-irradiated cells (Figure 2B).

### 2.3. TRF Exhibited Anti-Oxidative Effects against Oxidative Stress Induced by BL

**Figure 3:**
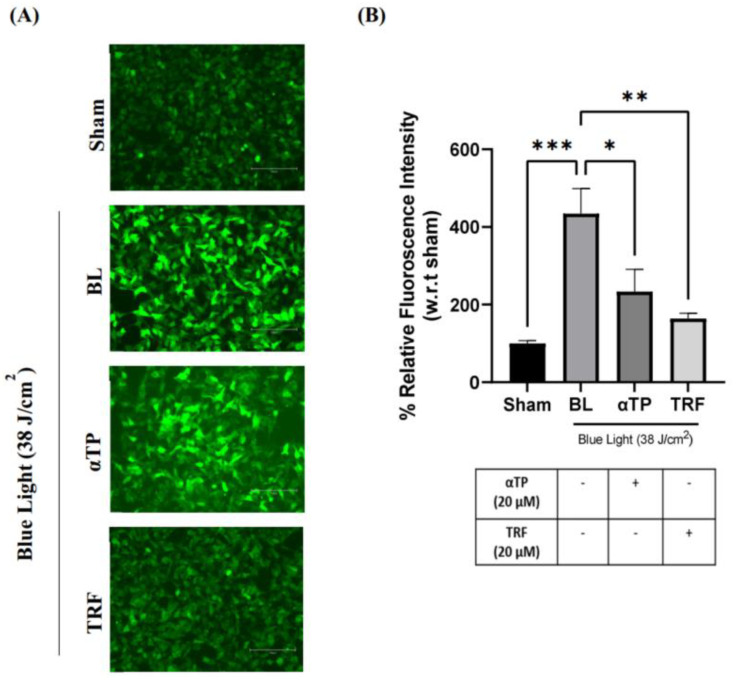
Presence of oxidative stress in B16-F1 melanocytes exposed to BL and treatment. (**A**) Fluorescence images of DCFDA as depicted by the green fluorescence. (**B**) Quantification of relative DCFDA fluorescence to represent intracellular ROS. Data shown are expressed as % of sham and expressed as mean ± SEM. *** *p* < 0.001, ** *p* < 0.01, and * *p* < 0.05 represent statistical significance. Scale bar: 100 µM.

Based on the findings presented in Section 2.1 and Section 2.2, we aimed to further investigate the presence of ROS in the cells, as they may have played a significant role in the induction of apoptosis. To examine the presence of oxidative stress in B16-F1 cells exposed to blue light, we used the 2′, 7′-dichlorofluorescin diacetate (DCFDA) fluorescent probe and captured images using fluorescence microscopy. We observed a stronger fluorescence signal in the cells irradiated with BL compared to the sham group (negative control group). However, treatment with αTP and TRF resulted in a reduction in the fluorescence signal, with TRF demonstrating a higher degree of reduction (Figure 3A). To determine the levels of intracellular ROS, we measured the fluorescence intensity and normalized it to the sham group. Both treatments showed protection against ROS, with TRF showing a higher anti-oxidative capacity compared to αTP (Figure 3B).

### 2.4. TRF Regulated BL-Induced Mitochondrial Membrane Potential Alterations

**Figure 4:**
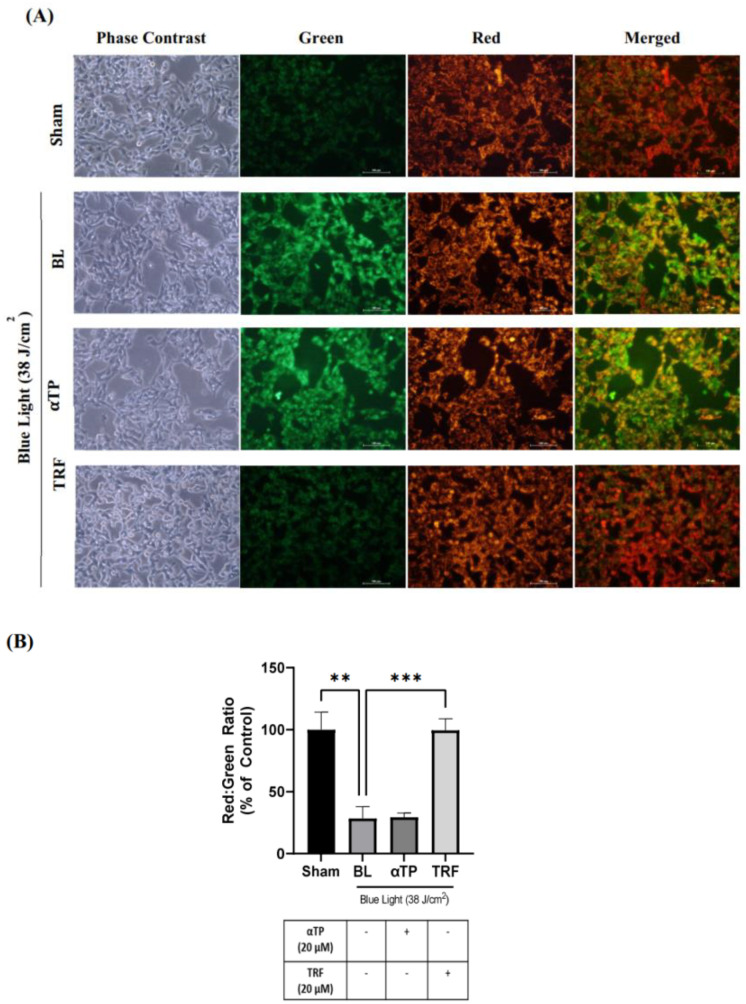
Mitochondrial membrane potential of B16-F1 melanocytes exposed to BL and treatments. (**A**) Fluorescence images of JC-1 dye. (**B**) Quantification of red/green fluorescence ratio to represent the polarization of the membrane potential. Data shown are expressed as % of sham and expressed as mean ± SEM. *** *p* < 0.001 and ** *p* < 0.01 represent statistical significance. Scale bar: 100 µM.

The presence of ROS in the cells could potentially compromise important cellular organelles such as the mitochondria. To assess the mitochondrial membrane potential, we utilized the JC-1 mitochondria staining kit. The presence of JC-1 monomers is indicated by green fluorescence and indicates a healthy mitochondrion, while the presence of JC-1 aggregates, represented by red fluorescence, indicates an unhealthy mitochondrion. Therefore, the ratio of red to green fluorescence can serve as an indicator of the membrane potential [19]. Upon BL induction, a stronger intensity of green fluorescence was observed compared to red fluorescence. However, treatment with TRF exhibited a rescuing effect on the mitochondrial membrane potential, as evidenced by a significant restoration of the ratio of red to green fluorescence (Figure 4A). As shown in Figure 4B, there was a significant reduction in the red/green fluorescence ratio following BL exposure, suggesting mitochondrial membrane depolarization. The ratio significantly improved back to baseline with TRF treatment, while this effect was not observed in the αTP group.

### 2.5. TRF Attenuated Cellular Death through Modulation of p38-MAPK Regulated Mitochondrial Apoptotic Pathway

**Figure 5:**
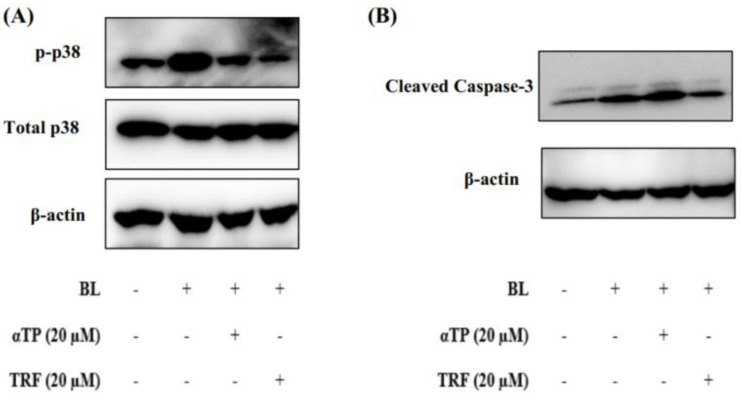
Protective effects of TRF against BL-induced p38 MAPK regulated mitochondrial apoptotic pathway. (**A**) Western blot analysis of p38 MAPK protein expression in BL exposed cells. (**B**) Western blot analysis showing activation of apoptotic marker upon BL induction.

Based on the results presented in Section 2.2 and Section 2.4, it can be theorized that mitochondrial dysfunction induced by BL may contribute to the observed cellular death. To understand the molecular mechanisms underlying this phenomenon, we studied the expression of proteins involved in the p38-MAPK regulated mitochondrial apoptotic pathway, namely p38 and caspase-3 [20]. Upon BL induction, there was an extensive activation of p38, as evidenced by increased phosphorylation. However, treatment with αTP and TRF resulted in a significant reduction in the levels of phosphorylated p38 (Figure 5A).

Additionally, we also investigated the expression levels of caspase-3 in B16-F1 cells exposed to BL. The activation of procaspase-3, indicated by the presence of cleaved caspase-3, was observed. Notably, treatment with TRF, but not αTP, significantly decreased the levels of cleaved caspase-3 (Figure 5B). These results align with the results obtained from the Annexin-V/PI and JC-1 assays, suggesting that TRF may protect the cells from mitochondria-mediated apoptosis by preserving the mitochondrial membrane potential.

### 2.6. TRF Prevented Pigmentation Induced by BL through Regulation of Tyrosinase Activity

**Figure 6:**
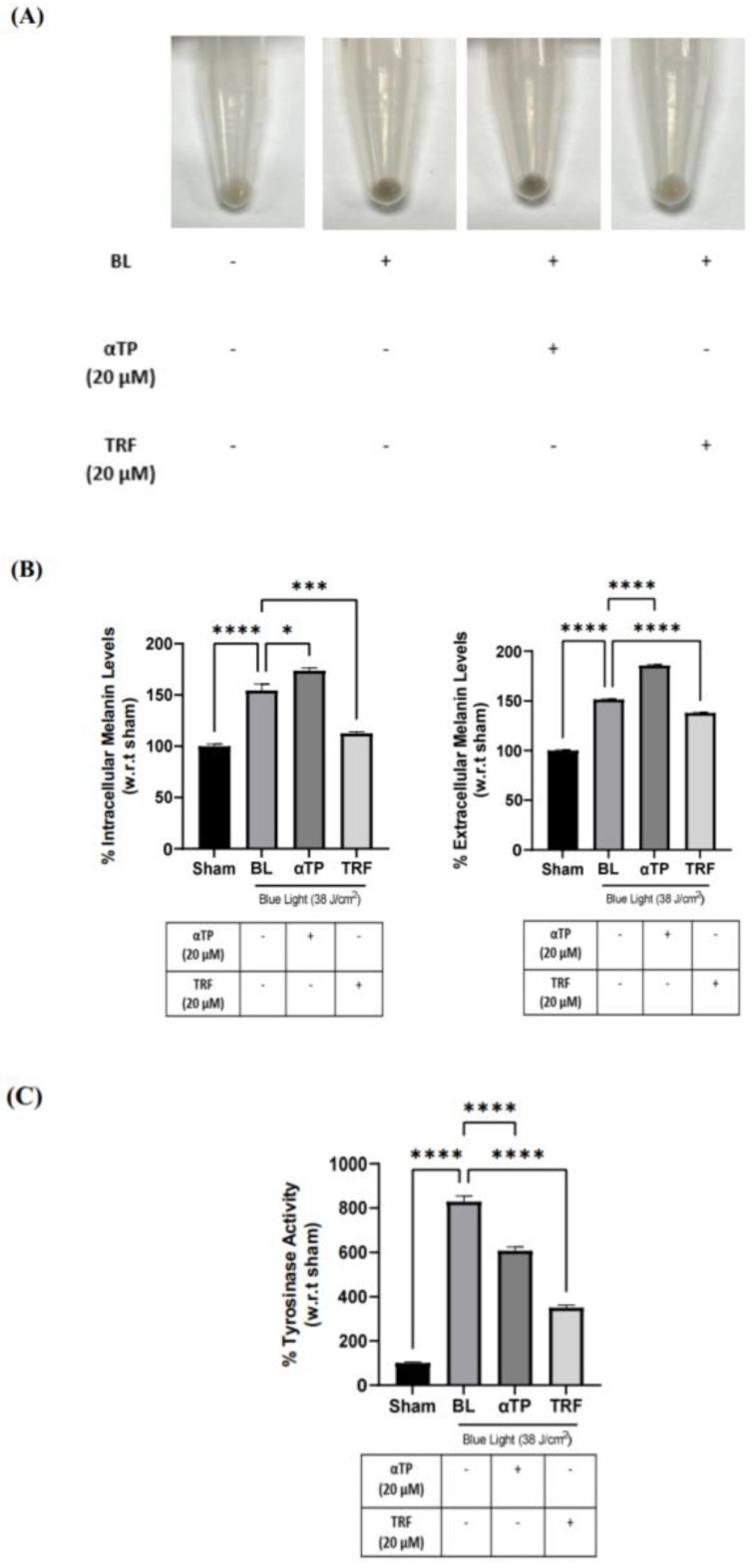
Pigmentation effects of BL on B16-F1 melanocytes and protective effects of TRF on melanogenesis process. (**A**) B16-F1 cell pellet colour after BL exposure and treatments. (**B**) Intracellular and extracellular melanin levels of cells induced by BL and treatment. (**C**) Tyrosinase activity in B16-F1 cells after BL and treatment. Data shown are expressed as % of sham and expressed as mean ± SEM. **** *p* < 0.0001, *** *p* < 0.001, and * *p* < 0.05 represent statistical significance.

To study the effect of BL on pigmentation and the rate of melanogenesis in B16-F1 cells, we conducted a melanin assay and measured tyrosinase activity. The pigmentation effect of BL on the B16-F1 cells was investigated by observing changes in cell pellet colour and measuring intracellular and extracellular melanin content. BL exposure induced a visible darkening of the cell pellets, which was reversed by treatment with TRF (Figure 6A). This finding was corroborated by the quantification of intra- and extracellular melanin levels, which showed a significant reduction in melanin levels in the TRF-treated groups (Figure 6B). To further elucidate the anti-pigmenting function of TRF, we assessed the tyrosinase activities of the BL-induced cells. There was a drastic increase in tyrosinase activity after BL exposure. While both αTP and TRF were able to decrease the activity level, TRF appeared to exhibit a more pronounced effect (Figure 6C).

### 2.7. TRF Exhibited Anti-Melanogenic Effects

**Figure 7:**
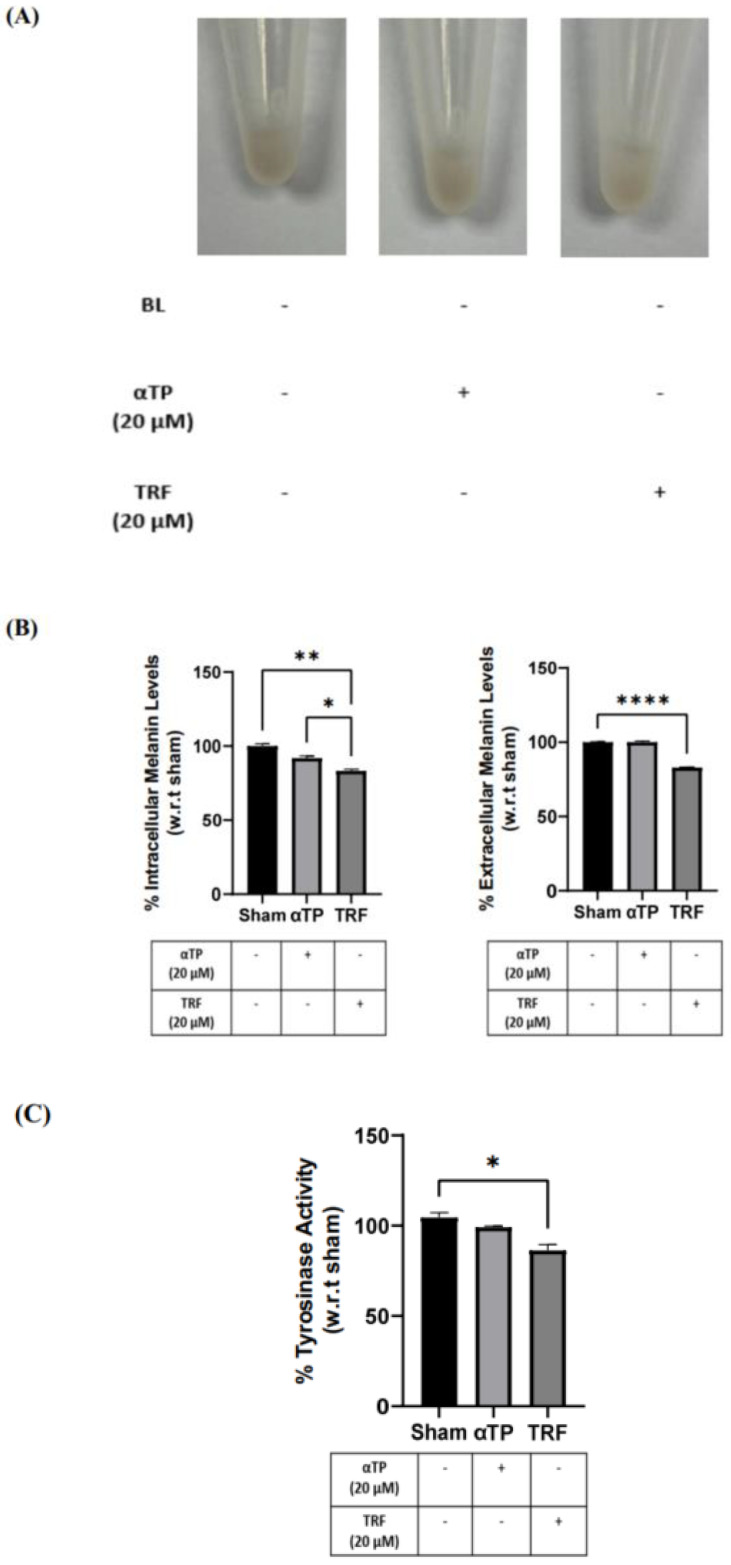
Pigmentation effects of αTP and TRF on the melanogenesis process. (**A**) B16-F1 cell pellet colour after treatments. (**B**) Intracellular and extracellular melanin levels of cells. (**C**) Tyrosinase activity in B16-F1 cells. Data shown are expressed as % of sham and expressed as mean ± SEM. **** *p* < 0.0001, ** *p* < 0.01, and * *p* < 0.05 represent statistical significance.

Based on the results obtained in Section 2.6, which exhibit the promising effects of TRF in preventing BL-induced hyperpigmentation, we aimed to further investigate the effects of TRF on pigmentation. To understand the mode of action behind the anti-pigmenting properties of TRF, we conducted the same melanin and tyrosinase assay on the B16-F1 cells. The B16-F1 cells were treated with αTP and TRF in the absence of BL induction. Similar to previous experiments, changes in cell pellet colour, melanin levels, and tyrosinase activity were measured. The TRF-treated cells exhibited a lighter pellet colour compared to the non-treated and αTP-treated cells (Figure 7A). This finding is supported by the quantification levels regarding intra- and extracellular melanin levels, along with the measurement of tyrosinase activity in the cells. Remarkably, TRF treatment resulted in a significant reduction in melanin levels and tyrosinase activity (Figure 7B). These findings demonstrate that TRF may exhibit anti-melanogenic properties by modulating tyrosinase activity in the cells.

## 3. Discussion

In our modern, fast-paced, and digitalized world, we are constantly surrounded by devices that emit BL, leading to an increase in the production of ROS. Melanin, a photoprotective and redox-active pigment, is produced in response to the increased oxidative stress caused by exposure to ROS. This pro-oxidative state triggers an upregulation of melanin production, resulting in skin darkening and hyperpigmentation [21]. Our study involved investigating the anti-oxidative, anti-apoptotic, and anti-melanogenic properties of TRF in BL-exposed B16-F1 melanocytes. Additionally, we also aimed to elucidate the molecular mechanisms underlying BL-induced apoptosis and pigmentation.

BL exposure is known to generate a significant amount of superoxide ROS, leading to cellular inflammation and oxidative stress. Under normal circumstances, our cells possess their own anti-oxidative systems that can eliminate the generated ROS. However, BL is known to oxidize the anti-oxidants produced by the cell, compromising the cell’s anti-oxidative capacity [22]. When a cell undergoes oxidative stress, it can result in irreversible damage. If the cell’s endogenous repair mechanisms are unable to rapidly address this damage, it can accumulate and ultimately lead to cell death [23]. In our study, we observed an increase in ROS levels in the B16-F1 cells exposed to BL. However, both treatments, αTP and TRF, were effective in reducing the ROS produced, with TRF exhibiting a more significant protective effect. T3s act as a potent anti-oxidant, scavenging free radicals more effectively than αTP due to their structural differences, which allow for better penetration. Furthermore, γT3s and TRF have been reported to possess various anti-oxidative properties, such as preventing a reduction in the expression of the enzyme superoxide dismutase (SOD) [24,25]. The regulation of SOD activity is crucial, as it is the only enzyme that exclusively interacts with superoxide, helping to keep the levels of ROS at a minimum in cells [26]. Therefore, our treatment with TRF might be beneficial in suppressing the formation of BL-induced superoxide, thereby protecting the cells from a pro-oxidative state.

The accumulation of ROS in cells can activate various signalling pathways, including apoptosis or necrosis-related pathways [27]. One of the major pathways involved in cellular apoptosis is the MAPK pathway. MAPK signalling cascades play a role in numerous physiological processes, such as proliferation, differentiation, inflammation, and apoptosis. Among the different MAPK subgroups, p38 is often activated in response to oxidative stress, inflammation, and DNA damage [28,29]. The activation of the p38 molecule is denoted by phosphorylation and can lead to either an intrinsic apoptotic pathway, activated by internal cell stresses, or an extrinsic apoptotic pathway, typically activated by external stimuli [30]. Oxidative stress within cells, as part of the intrinsic pathway, can contribute to the activation of pro-apoptotic factors, resulting in mitochondrial membrane permeabilization and alterations in mitochondrial membrane potential. This triggers a cascade of signalling events, including the release of the apoptogenic factor cytochrome c from the mitochondria into the cytoplasm. Cytochrome c in the cytosol then forms a complex with apoptotic protease activating factor-1 and caspase-9, further activating caspases such as caspase-3 and ultimately leading to cellular destruction [9,31].

In this study, we investigated the effect of TRF on the activity of the p38 MAPK molecule and the mitochondria-mediated apoptotic pathway. Our results showed that TRF effectively abolished the activation of the p38 molecule, improved the mitochondrial membrane potential, and reduced the expression of cleaved caspase-3. In a previous research study, Satyamitra et al. [32] reported that γT3, an isomer found in TRF, can suppress the activities of caspase-3 and caspase-7, but it does not affect the levels of cytochrome c. This suggests that γT3 may not directly rescue mitochondrial function. Additionally, a previous study substantiates our findings by showing that treatment with TRF can lower levels of ROS and MAPK proteins, which are precursors of oxidative stress [16]. Based on these observations, we theorize that TRF rescues B16-F1 cells exposed to BL due to its anti-oxidative nature, thereby terminating the downstream apoptotic signalling cascade. Further studies are warranted to elucidate the role and mode of action of TRF in the mitochondria-mediated apoptotic pathway.

Our study demonstrated that prolonged BL exposure increased the rate of melanogenesis in B16-F1 cells by modulating tyrosinase activity. Tyrosinase and dopachrome tautomerase are the key liming enzymes involved in melanogenesis, which is the process of skin pigmentation [33]. During melanogenesis, the formation of dopaquinone (DQ) is a rate-limiting step and is produced when L-tyrosine is oxidized by tyrosinase. The produced DQ then undergoes subsequent reactions to produce either pheomelanin or eumelanin, making tyrosinase a key enzyme in melanogenesis [34]. In addition to tyrosinase, melanin synthesis in melanocytes is regulated by microphthalmia-associated transcription factor (MITF), which is the master gene of melanocyte development and can be activated by various external stimuli, including the MAPK signalling pathway [35].

In our study, TRF exhibited anti-melanogenic properties by reducing the levels of intra- and extracellular melanin produced upon BL induction. This could be attributed to the anti-oxidative functions of TRF. Furthermore, our results also established that TRF inhibited melanogenesis by modulating tyrosinase activity, suggesting that TRF may have a direct effect on melanin synthesis. Although the expression of MITF would be crucial in discussing the melanogenic pathways, our findings showed a significant decrease in tyrosinase activity with TRF treatment. Considering that tyrosinase expression is tightly regulated by MITF, it is plausible to consider that TRF may also exert an inhibitory effect on MITF expression [36].

Our experimental design provided quantitative evidence that supports our hypothesis and enhances our understanding of the effects of BL on melanocytes. To the best of our knowledge, this is the first study to demonstrate the efficacy and protective effects of TRF against BL-induced oxidative stress and melanogenesis, particularly in comparison with αTP, which is a more commonly used active ingredient in the skin care market. However, we acknowledge that there are certain limitations to this study. To gain a more comprehensive understanding of the mode of action of TRF, further molecular research is needed. Specifically, the use of inhibitors targeting various signalling pathways, such as p38-MAPK, which regulates mitochondrial apoptosis and melanogenesis, would be beneficial in elucidating the precise mechanism by which TRF exerts its effects. This would provide deeper insights into the molecular pathways involved in the protective actions of TRF against BL-induced oxidative stress and melanogenesis. Moreover, to validate the protective effects of TRF on BL-induced skin damage, additional randomized controlled clinical trials are also needed.

## 4. Materials and Methods

For our study, we conducted various assays to investigate the protective effects of TRF on BL-exposed melanocytes. Section 4.1 and Section 4.2 list the cell culture reagents used and treatment preparation. Section 4.3 outlines the BL panel parameters. Section 4.4 and Section 4.5 describe the MTT proliferative assay and the apoptosis assay, the latter of which was carried out via flow cytometry. Section 4.6 and Section 4.7 describe the fluorescence imaging analysis for ROS and mitochondrial membrane potential. Section 4.8 and Section 4.9 detail the melanogenesis rate and tyrosinase activity measurements. Section 4.10 explains the Western blotting protocol, and Section 4.11 states the statistical analysis protocols utilized for this study.

### 4.1. Cell Culture and Reagents

Murine melanoma B16-F1 cell line (CRL-6323) was purchased from American Type Culture Collection (ATCC, Manassas, VA, USA). The cells were cultured in Dulbecco’s modified Eagle’s Medium (DMEM; Nacalai Tesque Inc., Kyoto, Japan) supplemented with 10% (*v*/*v*) foetal bovine serum (HyClone Laboratories, Logan, UT, USA) and 1% (*v*/*v*) penicillin G (100 U/mL) and streptomycin (100 µg/mL) (gibco, Thermo Scientific, Waltham, MA, USA). The cells were grown and maintained at 37 °C with 5% CO_2_ and humidity. Monoclonal antibodies against phospho-p38, total p38, and cleaved caspase 3 were obtained from Cell Signaling Technology (Danvers, MA, USA). A monoclonal antibody against β-actin was obtained from Santa Cruz Biotechnology (Santa Cruz, CA, USA). Phosphate buffered saline (PBS), 0.05% trypsin, and phenol red-free DMEM were purchased from Gibco (Thermo Scientific). Dimethyl sulfoxide (DMSO) was purchased from Kanto Chemical Co. (Tokyo, Japan). Methanol and absolute ethanol were purchased from Fisher Scientific (Thermo Scientific). MTT, αTP, synthetic melanin, l-3,4-dihydroxyphenylalanine (L-DOPA), bovine serum albumin (BSA), DCFDA, and JC-1 kit were obtained from Sigma-Aldrich (St. Louis, MO, USA). TRF with a purity of ≥95% was supplied by Davos Life Science Sdn Bhd (DavosLife E3, Petaling Jaya, Malaysia).

### 4.2. Natural Extract and Cell Treatment

The TRF and αTP stock solution were prepared in absolute ethanol at a concentration of 100 mM and stored at −20 °C. The stocks were then diluted in complete DMEM or phenol red-free DMEM to a final concentration of 20 µM for each treatment. The B16-F1 cells were incubated with the respective treatments for 24 h before irradiation, during the irradiation, and after irradiation where necessary.

### 4.3. Cell Irradiation

The B16-F1 cells were subjected to artificial BL irradiation with the aim of inducing oxidative stress and melanin synthesis within the melanocytes. The BL source was a panel of LED light bulbs (15 × 15) with a power of 3.6 mW/cm^2^, which emitted light at 465 nm (HQRP, Harrison, NJ, USA) and were placed in a customized WCI-40 CO_2_ incubator (Bio Laboratories Pte Ltd., Singapore). The B16-F1 cells were exposed to a BL irradiation dosage of 38 J/cm^2^, which is equivalent to 3 h of in vitro BL exposure. Immediately after irradiation, fresh media with the respective treatments were added, and the cells were maintained in the CO_2_ incubator.

### 4.4. MTT Cell Viability Assay

The B16-F1 cells were cultured in the individual wells of a 96-well plate at a density of 5 × 10^3^ cells/well for 24 h. The cell viability of the cells was measured 24 h after BL irradiation. MTT solution (0.5 mg/mL) was added into each well for 2 h and incubated at 37 °C with 5% CO_2_ and humidity. The formazan crystals were dissolved in 200 µL of DMSO, and the absorbance was measured at 595 nm using the Enspire^®^ Multimode Plate Reader (Perkin Elmer, Waltham, MA, USA).

### 4.5. Annexin-V Assay by Flow Cytometry

Cellular death was assessed by using the Dead Cell Apoptosis Kit with Annexin V (Thermo Scientific) according to the manufacturer’s protocol. The cells were cultured in 60 mm Petri dishes at a density of 2 × 10^5^ cells/dish for 24 h. At 24 h after BL exposure, the cells were harvested by trypsinization, washed twice in ice cold PBS, and resuspended in 1× annexin-binding buffer at a concentration of 1 × 10^6^ cells/mL. The samples were then used for flow cytometry analysis using the Accuri™ C6 Plus System (Becton Dickson, Franklin Lakes, NJ, USA).

### 4.6. Intracellular ROS Assay

The B16-F1 cells were cultured in 6-well plates at a density of 0.8 × 10^6^ cells/well for 24 h. Immediately after BL irradiation, the cell culture medium was aspirated, and the cells were incubated with 10 µM of DCFDA dye for 30 min in the dark at 37 °C. The cells were then washed twice in PBS, and microscopic images were obtained using the EVOS M5000 Imaging System (Thermo Scientific) with a green excitation filter. Fluorescence intensity was then analysed using ImageJ version 1.8.0_172 (National Institutes of Health, Bethesda, MD, USA), and data were normalized with respect to the respective sham values.

### 4.7. Mitochondrial Membrane Potential

To assess mitochondrial membrane potential, the JC-1 assay was conducted. The B16-F1 cells were cultured in 60 mm Petri dishes at a density of 2 × 10^5^ cells/dish for 24 h. Immediately after BL irradiation, the cell culture medium was aspirated, and the cells were incubated with 5 ug/mL of JC-1 dye for 20 min in the dark at 37 °C. Subsequently, the cells were washed twice in incomplete DMEM. Microscopic images were obtained using an inverted microscope (Eclipse TE2000-U, Nikon, Minato City, Tokyo, Japan) with orange–red and green filters emitted from a Nikon Intensilight C-HGFI. Images were captured using a Nikon Digital Sight DS-U2. Fluorescence intensity was analysed using ImageJ version 1.8.0_172 (National Institutes of Health), and data were normalized with respect to the respective sham values.

### 4.8. Intracellular and Extracellular Melanin Content

The B16-F1 cells were cultured in 60 mm Petri dishes at a density of 2 × 10^5^ cells/dish for 24 h. After 24 h, the cells were harvested by scraping, and the cell culture medium were collected. The cells were then incubated at 60 °C at 1 h in 1 M NaOH and vortexed to solubilize the melanin pigments. Following that, the cell suspensions and culture medium were centrifuged at 1500× *g* rpm for 15 min. The absorbance was then measured at 405 nm using the EnSpire^®^ Multimode Plate Reader (Perkin Elmer) and compared to a standard curve prepared using synthetic melanin. Thereafter, the intracellular and extracellular melanin contents were determined based on absorbance/µg of protein. The protein content was determined using the DC protein assay kit (Bio-Rad, Hercules, CA, USA).

### 4.9. Tyrosinase Activity

Cellular tyrosinase activity was determined as reported by Lee et al. with slight modifications [37]. The cells were cultured in 60 mm Petri dishes at a density of 2 × 10^5^ cells/dish for 24 h. At 24 h after irradiation and treatment, the cells were washed with PBS and collected with lysis buffer. The cells were then ruptured by freezing for 30 min in −80 °C, followed by thawing on ice. Afterwards, the lysate was clarified by centrifugation at 13,000× *g* rpm for 20 min at 4 °C. The protein content was determined using the DC protein assay (Bio-Rad). An equal amount of protein was then added into each well of a 96-well plate, followed by 10% of 2 mg/mL L-DOPA prepared in phosphate solution. The well plate was then incubated at 37 °C for 1 h, and the absorbance of the mixture was measured using the EnSpire^®^ Multimode Plate Reader (Perkin Elmer).

### 4.10. Western Blot

Whole lysates were collected by resuspending the cell pellets with RadioImmunoPrecipitation Assay (RIPA) lysis buffer containing 50 mM Tris-HCl pH 8.0, 150 mM sodium chloride, 1 mM ethylenediaminetetraacetic acid, 1% *v*/*v* nonidet P-40, 0.5% *v*/*v* sodium deoxycholate, and 0.1% *v*/*v* sodium dodecyl sulfate supplemented with protease and phosphatase cocktail inhibitors (Roche, Basel, Switzerland). The protein concentration was determined using the DC Protein Assay Kit (Bio-Rad) according to the manufacturer’s instructions, and protein standards were prepared using BSA. The protein samples were then prepared and loaded onto polyacrylamide gel electrophoresis at 20 mA for 1 h using the Mini-PROTEAN 3 Cell (Bio-Rad). The membrane was then blocked with 10% *v*/*v* non-fat milk in Tris-buffered Saline (TBS) supplemented with 0.1% *v*/*v* Tween-20 (TBS-T). Subsequently, the blots were washed twice with TBS-T and then incubated with the corresponding antibodies at 4 °C overnight. The blots were then washed thrice with TBS-T to remove the unbound primary antibodies before exposure to IgG-HRP-conjugated secondary antibodies in 5% *v*/*v* non-fat milk in TBS-T for 1 h at room temperature (25 °C). The unbound secondary antibodies were removed by being washed thrice with TBS-T. Chemiluminescent protein bands were then visualized by ECL Select Western Blot Detection Reagent (Amersham, Piscataway, NJ, USA) using the ChemiDoc MP Imaging System, and ImageLab TM software version 6.1 (both from Bio-Rad) was used for densitometric analysis.

### 4.11. Statistical Analysis

All results are expressed as the mean ± standard error of the mean (SEM), and the data obtained nwere statistically evaluated via a one-way analysis of variance (ANOVA), followed by Tukey’s multiple comparisons test carried out using GraphPad Prism 9.4.1 (GraphPad Software, San Diego, CA, USA). A *p* value of <0.05 (*) was considered to be statistically significant.

## 5. Conclusions

**Figure 8:**
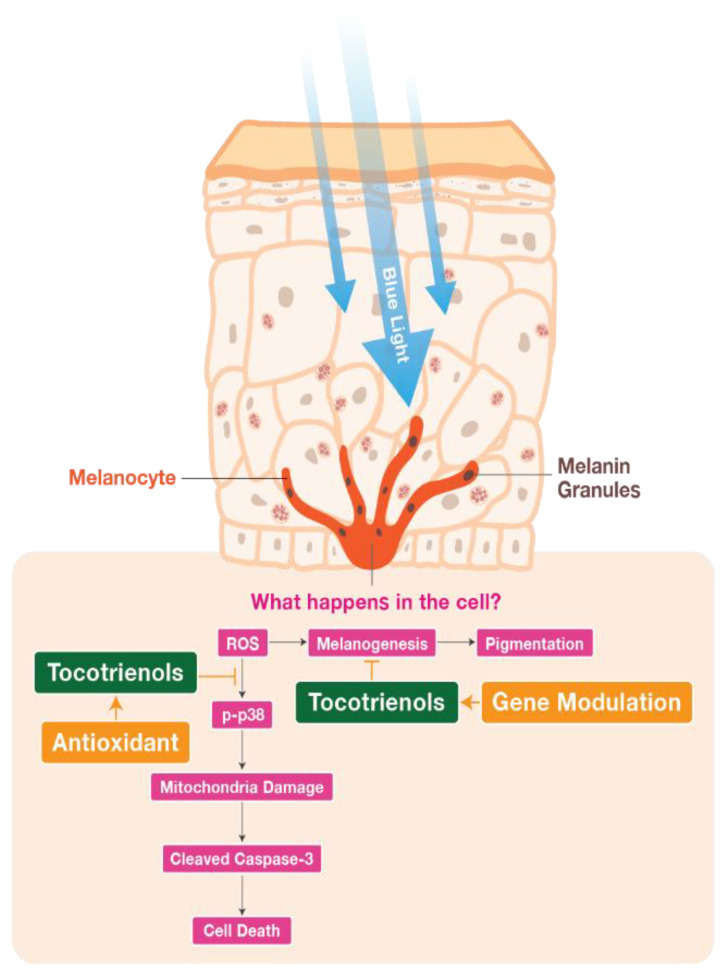
Schematic diagram illustrates the suggested mechanisms underlying the anti-oxidative and anti-melanogenic effects of TRF in BL-induced cellular stress and melanogenesis in B16-F1 cells. TRF effectively reduces the ROS production induced by BL, thereby inhibiting the activation of the p38 MAPK molecule. This inhibition subsequently protects the mitochondrial membrane potential of the cells and prevents cell death. Additionally, TRF demonstrates the ability to ameliorate the hyperpigmentation effects induced by BL through its ROS-protecting properties and modulation of tyrosinase activity in B16-F1 cells.

Exposing the skin to BL for a prolonged period of time can disrupt skin health by generating ROS and causing hyperpigmentation, which can accelerate skin ageing [38]. BL also has detrimental effects on mitochondrial health, leading to a decline in cell function and, eventually, cell death [39]. Our study demonstrated that BL exposure induced cell death and hyperpigmentation in melanocytes. However, treatment with TRF was able to protect melanocytes from cell death and hyperpigmentation, potentially through the regulation of ROS and mitigation of mitochondria damage. TRF treatment also provided anti-pigmentation properties by regulating tyrosinase activity. In summary, our results indicate that TRF exhibits more robust protective effects against BL-induced damage in melanocytes compared to αTP through a combination of anti-oxidative and anti-melanogenic mechanisms (Figure 8). These findings support the potential of TRF as an active agent for protecting melanocytes from BL-induced damage. Future research, including human clinical trials, are needed to validate the effects of TRF on human skin.

## Data Availability

No new data were created or analysed in this study. Data sharing is not applicable to this article.
